# Antiviral immunity and nucleic acid sensing in haematopoietic stem cell gene engineering

**DOI:** 10.1038/s41434-020-0175-3

**Published:** 2020-07-13

**Authors:** Francesco Piras, Anna Kajaste-Rudnitski

**Affiliations:** San Raffaele Telethon Institute for Gene Therapy, IRCCS San Raffaele Scientific Institute, Milan, Italy

**Keywords:** Haematopoietic stem cells, Innate immunity

## Abstract

The low gene manipulation efficiency of human hematopoietic stem and progenitor cells (HSPC) remains a major hurdle for sustainable and broad clinical application of innovative therapies for a wide range of disorders. Given that all current and emerging gene transfer and editing technologies are bound to expose HSPC to exogenous nucleic acids and most often also to viral vectors, we reason that host antiviral factors and nucleic acid sensors play a pivotal role in the efficacy of HSPC genetic manipulation. Here, we review recent progress in our understanding of vector–host interactions and innate immunity in HSPC upon gene engineering and discuss how dissecting this crosstalk can guide the development of more stealth and efficient gene therapy approaches in the future.

## Introduction

Haematopoietic stem cell (HSC) gene therapy can potentially provide life-long therapeutic benefits in all progeny cells upon ex vivo gene engineering, rendering them attractive targets for the cell and gene therapy of a number of congenital disorders, infectious diseases, and cancers [1]. The large progeny of a single HSC dictates the use of stable gene delivery systems to ensure long-term genetic changes in blood and immune cells. Therefore, the use of integrating recombinant viral systems such as ɣ-retroviruses (ɣRV) and lentiviruses (LV) has thus far dominated this field. Recent progress in genome editing technologies based on programmable nucleases such as zinc-finger nucleases (ZFN) and the clustered regularly interspaced short palindromic repeat (CRISPR)-associated nuclease Cas9 is also opening alternative pathways for gene therapy [2]. In this context, the non-integrating adeno-associated vectors (AAV) have become a widely exploited vehicle of donor DNA required for homology-directed repair (HDR) in HSC [3]. Single-strand and double-strand oligodeoxynucleotides (ODN) are also emerging as effective means to deliver donor templates for HDR in several clinically relevant settings, including in HSC [4].

Current HSC gene therapy protocols are based on the modification of bone marrow (BM)-or mobilized peripheral blood (mPB)-derived CD34^+^ cells, enriched in HSC but containing also a large fraction of more differentiated progenitor cells, termed all together haematopoietic stem and progenitor cells (HSPC). Despite evident success of recent LV-based clinical trials for patients affected by primary immunodeficiencies (PID), haemoglobinopathies, or inborn errors of metabolism, improving HSPC transduction efficiency remains a high priority goal for the field as these cells are particularly refractory to gene transfer and significant variability in the therapeutic outcome among patients has been observed [5–7]. Indeed, high vector doses, multiple rounds of transduction and prolonged ex vivo culture are still required to reach clinically relevant gene marking and editing levels. This imposes costly large-scale vector production and potentially compromises HSPC preservation in culture as increasing evidence shows that cultured HSPC progressively lose engraftment potential through cell cycle progression and loss of adhesion molecules, thus impairing their homing into the niche and driving lineage commitment and differentiation [8–11]. Of note, ex vivo culture is usually even longer in the context of gene editing protocols, since HDR occurs only when cells are actively cycling [12].

HSPC are responsive to innate immune cues such as TLR ligands [13], foreign nucleic acids [14, 15], and IFN-inducing viruses [16] with potentially harmful biological consequences. Because all current and emerging gene transfer and editing technologies are bound to expose them to exogenous nucleic acids and most often also to viral vectors, we believe that host antiviral factors and nucleic acid sensors play a pivotal role in the efficacy of HSPC genetic manipulation. In agreement, a number of experimental evidences indicate that strategies aimed at modulating innate immune activation may improve gene engineering efficiency. In this review, we will highlight recent progress in our understanding of vector–host interactions and innate immunity in HSPC and discuss how dissecting this crosstalk can guide the development of more stealth and efficient gene therapy approaches in the future.

## Nucleic acid sensing in HSPC gene therapy

As the first line of host defence against pathogens, the innate immune system employs a limited number of germline-encoded receptors called pattern-recognition receptors (PRR) to detect and respond to the presence of pathogens. PRR recognize conserved molecular structures known as pathogen-associated molecular patterns (PAMP) that are essential for the life cycle of the pathogen. Many PAMP, such as LPS, peptidoglycans, and flagellin, are found in microbes but not in the host, allowing the host to distinguish non-self from self through PRR. One apparent exception is the detection of pathogen-derived nucleic acids. Many of these innate sensing mechanisms are likely subject to selective pressure due to continuous exposure of the host to invading pathogens in an evolutionary arms race often termed the Red Queen conflict [17]. As a result, mammalian cells have developed over time a plethora of potentially redundant immune sensing pathways. Conversely, viruses continue to evolve new means to evade these host responses, leading to genetic tug-of-wars between host and pathogen that tend to reach a dynamic equilibrium of mutual adaptation.

### Toll-like receptors

Among PRR, the family of Toll-like receptors (TLR) have been the most extensively studied. TLR localize on the cellular and endosomal membrane and monitor the lumen of endosomes and lysosomes. They detect various forms of nucleic acids from bacteria and viruses, and are mostly express on macrophages and dendritic cells. There are ten different TLR identified in humans. TLR1, 2, 4, 5, 6, and 10 are expressed at the cell surface and mainly recognize hydrophobic molecules unique to microbes and not produced by the host. In contrast, TLR3, 7, 8, and 9 are located almost exclusively in endosomal compartments and are specialized in nucleic acid recognition. TLR9 recognizes unmethylated CpG-rich DNA in endosomes, thereby detecting DNA entering through the endosomal or autophagocytic route [18]. TLR7/8 are highly homologues and recognize single stranded RNA, while TLR3 is activated by dsRNA and can be engaged by the synthetic dsRNA poly(I:C) in vitro. Human CD34^+^ cells express TLR3, TLR4, TLR7, TLR8, and TLR9 [13]. TLR7 and TLR8 activation in vitro causes myeloid progenitor skewing [19] while TLR3 stimulation by poly(I:C) leads to a strong IFN-stimulated genes (ISG) induction and apoptosis in HSPC [20]. The expression of TLR on HSPC suggests that they are competent to detect infection directly and respond with immediate production of immune effector cells through skewed differentiation. In agreement, LPS, the major ligand for TLR4, induces HSPC proliferation and mobilization out of the BM [21].

**Fig. 1:**
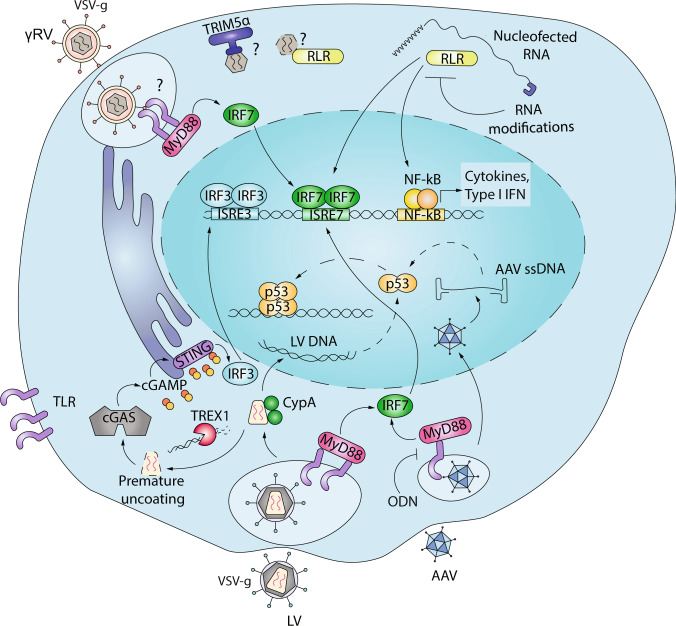
Nucleic acid sensing of genetic engineering. LV and AAV have been reported to activate TLR signalling (TLR9 and 7) at the endosomal level, thus leading to Myd88-mediated IRF activation. G-rich ODN can suppress TLR9-mediated innate immune activation. Upon entry, LV and γRV can be recognized by cytosolic RIG-I like receptors (RLR) or cGAS. LV usually evade this response by exploiting cellular factors such as CypA or TREX1 to prevent premature uncoating or excessive production of RT intermediates that could lead to production of cGAMP by cGAS and STING activation followed by IRF3 and NF-kB-mediated Type I IFN production. Nucleofected RNA during gene editing can activate RLR and induce ISG. RNA modification with 5-methylcytosine and pseudouridine prevents this activation. Upon nuclear entry, LV and AAV genome trigger p53-mediated DDR in human HSPC (see Fig. 2 for details).

Because lenti- and retroviral vectors reverse transcribe their RNA genome into double-stranded DNA in the cytoplasm of transduced cells, innate nucleic acid sensors may pose a particular problem for these vectors. Moreover, while HIV-1 enters the cell through membrane fusion, VSV-g pseudotyped vectors will undergo endocytosis, potentially exposing them to the several endocytic immune sensor including TLR (Fig. 1). Nevertheless, initial studies conducted in a human B cell line showed remarkably low impact of LV transduction on cellular gene expression [22]. Similarly, we have observed that LV transduction remains remarkably stealth and does not trigger TLR-mediated transcriptional programmes in human HSPC [23]. Instead, TLR7, TLR3, and TLR9 have been shown to recognize LV in murine plasmacytoid dendritic cells (pDC) leading to their maturation [24]. In vivo administration of LV to target hepatocytes for haemophilia gene therapy induced an innate immune response in the liver that was dependent on TLR and non-TLR signalling [25]. Importantly, blocking this innate immune response increased liver transduction efficiency [25] (Fig. 1).

Innate immune response to AAV is usually reported to be low, when compared to immune responses to Adenoviral vectors [26]. Nevertheless, activation of TLR9 has been implicated in eliciting a CD8^+^ T cell response towards the transgene product upon AAV transduction [27]. This TLR9 activation is not dependent on the serotypes or the transgene [28] and is followed by the induction of type I IFN in pDC in vitro, thus contributing to the development of adaptive immune responses towards the transgene or the AAV capsid [27]. Interesting differences in innate sensing of single stranded (ssAAV) and self-complementary AAV (scAAV) have been reported. While ssAAV induce a mild and transient IFN response that decays around 6 h after in vivo transduction, scAAV activate higher levels of IFN that lead to the production of systemic inflammatory cytokines [28] (Fig. 1). These observations suggest the presence of two different mechanisms of nucleic acid recognition depending on the vector genome structure and replication.

### RNA sensors

Innate immune receptors detecting RNA are referred to as RIG-I like receptors (RLR). The three central members of the mammalian RLR family, retinoic acid-inducible gene I (RIG-I), melanoma differentiation factor 5 (MDA5), and laboratory of genetics and physiology 2 (LGP2), are found in the cytosol of most cell types and can be strongly induced by IFN in a positive-feedback loop after viral detection [29]. RIG-I detects RNA with a triphosphate (PPP) and blunt-ended base-paired region of about 20 nucleotides at the 5′ end, allowing it to discriminate virus from self [30]. MDA5 detects dsRNA, normally absent in uninfected cells [31]. Once activated, both RIG-I and MDA5 induce the polymerization of MAVS on the mitochondrial surface, leading to a strong IRF3 activation by phosphorylation and the induction of type I IFN genes [32]. Much less is known of the nature of RNA that might bind to LGP2, the third RLR family member, but some in vitro studies have shown that LGP2 can cooperate with MDA5 for proper RNA recognition and signalling [33]. LV and γRV exploit the host cell machinery to 5′-cap their ssRNA genomes thus avoiding RIG-I mediated recognition [34]. Nevertheless, we have observed that γRV transduction still induces an ISG response in human HSPC [23]. Interestingly, this induction was unaffected by RT inhibition suggesting involvement of RNA sensing. Alternatively, structural components such as the viral capsid could potentially be detected by host factors such as human TRIM5α shown to bind N-MLV capsid protein p30 [35] and to mediate innate immune signalling further amplified by retroviral infection and interaction with the capsid lattice [36]. Distinct nuclear import kinetics may also explain differences in LV and γRV sensing in HSPC [37]. As γRV require the cell to be cycling to enter the nucleus, it is possible that the viral replication intermediates remain exposed for a prolonged period of time to the cytosolic sensors in HPSC as compared to LV with active nuclear import. RNA sensing has been shown to occur in the context of gene editing in human HSPC when zinc-finger nucleases are nucleofected as mRNA. Importantly, this sensing was significantly reduced by modifying the in vitro transcribed RNA with 5-methylcytosine and pseudouridine nucleotide modifications that are commonly found in cellular RNA [38] (Fig. 1).

### DNA sensors

Besides pathogen-associated RNA, the presence of exogenous DNA is a major danger signal within cells. As the chemical composition of the core DNA molecule is identical for mammalian cells and microorganisms, the principle of recognition is based mainly on subcellular localization of DNA sensors in this case. Mammalian genomic DNA is localized in the nucleus. Thus, most of the DNA sensors are found in other cellular compartments such as endosomes for TLR9 [39]. DNA sensors such as cyclic GMP-AMP (cGAMP) synthase (cGAS) and absent in melanoma 2 (AIM2) act mainly within the cytoplasm and detect DNA in a sequence-independent manner [40–42]. Upon DNA binding by cGAS, synthesis of the cyclic dinucleotide 2′,3′-cGAMP is initiated. cGAMP binds with high affinity to the stimulator of IFN genes (STING), a crucial signalling adaptor for type I IFN induction (Fig. 1). Once activated by cytosolic DNA signalling, STING undergoes a relocalization from the endoplasmic reticulum to the Golgi complex and assembles into punctate structures resulting in the phosphorylation of IRF3 and activation of the downstream pathway [43]. More recently, several reports identify cGAS as a nuclear protein but its role in DNA sensing and innate immune activation remains to be fully elucidated [44–47].

**Fig. 2:**
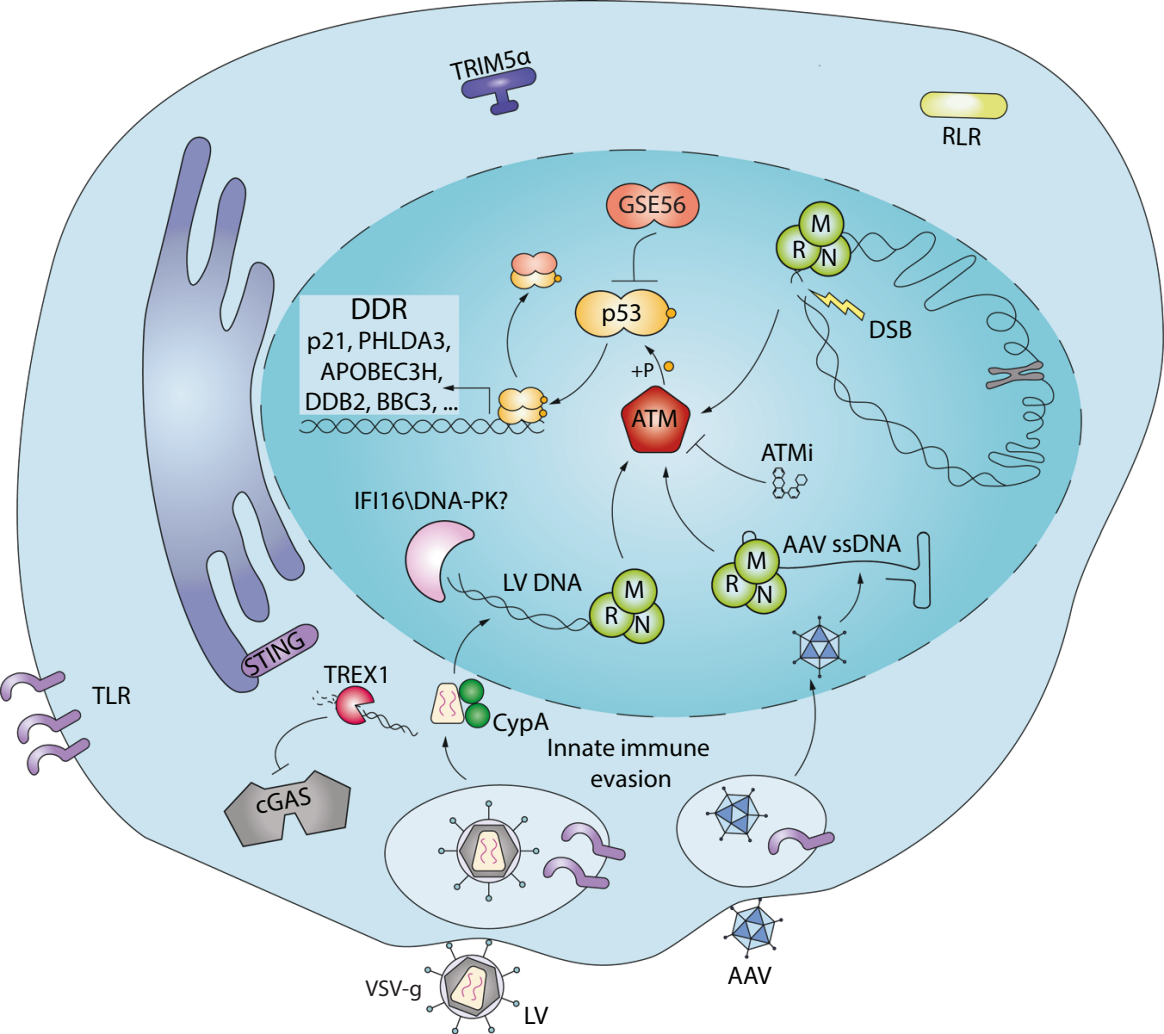
LV and AAV induce DDR in human HSPC. Upon transduction of human HSPC, LV and AAV form DNA intermediates that directly reach the nucleus evading innate immune radars. Once the LV and AAV genomes are in the nucleus the MRN complex, IFI16, DNA-PK or other factors sense the presence of viral DNA and activate an ATM-depednent DDR. ATM phosphorylates p53, which transcriptionally activates its downstream targets causing cell cycle arrest (p21), apoptosis (PHLDA3), and/or DNA repair. A similar DDR occurs also upon CRISPR/Cas9- or ZFN-induced DSB. The induction of this response can be inhibited by using ATM inhibitors during transduction or by transiently expressing a dominant negative form of p53 (GSE56).

Cytosolic cGAS has been shown to recognize VSV-G pseudotyped HIV-1 and other retroviral DNA in the THP-1 cell line, monocyte-derived macrophages (MDM) and monocyte-derived dendritic cells (MDDC) when additional blocks to replication are removed [48, 49]. Viral capsid stability can also impact innate immune sensing as altering favourable host-capsid interactions leads to premature uncoating [50] and exposure of viral intermediates in the cytosol where they can be recognized and trigger type I IFN in MDM [51] (Fig. 1). Of note, when some of these critical host interactions are prevented, LV still fail to elicit type I IFN responses in human HSPC [52]. It is possible that some additional mechanisms to prevent innate immune activation are in place specifically in HSPC. Also the gamma-interferon inducible protein (IFI16) has been shown to sense and control HIV in human macrophages [53]. IFI16 contains domains that are competent for DNA binding and proteins interaction [54]. It was discovered as a nuclear DNA sensor of cytomegalovirus [55], but can recognize HIV DNA intermediates in the cytosol and contribute to viral control in MDM [53]. Recognition of HIV incomplete reverse transcript by IFI16 has also been linked to HIV pathogenesis, leading to CD4^+^ T cells death by pyroptosis [56, 57]. Whether IFI16 plays a role in DNA sensing in HSPC remains to be addressed (Fig. 2). Given their artificial origin, ssODN and naked nucleic acids, have been much less studied in this context. However, as they are emerging as useful tools for gene editing, it will become increasingly relevant to understand how target cells react or not to these exogenous nucleic acids.

## DNA damage responses

A complex interplay between innate immune sensing and DNA damage is emerging. Cells that undergo DNA damage can activate innate immune signalling by non-canonical pathways involving IFI16 and STING [58, 59] and nuclear cGAS has been reported to play a role in DNA repair and tumorigenesis [44]. Conversely, viral infections or transduction can lead to DNA damage response (DDR) due to double-strand breaks (DSB) during the viral integration process in HeLa cells [60, 61] or to the presence of ssAAV genome in U2OS cells [62–64]. HIV and AAV have evolved accessory proteins that can exploit host factors involved in DNA damage sensing and repair for efficient viral replication, although some controversy exists, since blocking DDR does not inhibit HIV integration [65] but can still alter viral replication and transduction [66]. Interestingly, the canonical DDR protein DNA-dependent protein kinase (DNA-PK) has been very recently shown to mediate STING-independent DNA sensing and type I IFN activation in human cells [67]. Sensors of DNA damage can thus be considered as alternative innate immune radars that act mainly through p53 activation but can also trigger type I IFN responses.

DNA damage sensors respond to a wide range of DNA lesions with remarkable sensitivity as even a single DSB in the double helix can induce DDR and, if left unresolved, cell death [68]. Initial recognition of damaged DNA usually relies on Mre11-Rad50-Nbs1 (MRN) for DSB [69], while other sensors like XPC-RAD23-CETN2 can recognize UV-induced damage [70]. Once the DNA damage is recognized, at least one of the three kinases ataxia-telangiectasia mutated (ATM), ataxia telangiectasia and Rad3 related (ATR), or DNA-PK transduce and amplify the signal into a proper DDR, extensively reviewed elsewhere [71]. ATM and ATR have been shown to potentially phosphorylate more than 700 targets, including the DDR master regulator p53 that acts as a transcription factor to induce expression of DDR effector proteins leading to cell cycle arrest, DNA repair or apoptosis [71–73] (Fig. 2). For AAV, DDR signalling appears to aid viral replication, although it is unclear to which degree DDR results from replication stress and impacts cell cycle and nuclease activity of viral nonstructural proteins [74]. During productive AAV replication with an adenovirus as helper, DDR is activated through DNA-PK [75], while with HSV-1 as helper, mainly ATM is involved [76]. MRN can directly bind the AAV genome on the inverted terminal repeat (ITR) and impair replication in HeLa cells [77] (Fig. 2). As a potential antagonizing strategy, adenoviral oncoproteins can reorganize and degrade members of the MRN complex to enhance viral replication [78]. Interestingly, adenoviral and HSV-1 proteins can also block the recently identified function of DNA-PK as an alternative DNA sensor and innate immune activator [67]. Many of these data have been collected in cell lines, which often harbour compromised immune sensing and DDR pathways. Therefore, further studies aimed at confirming these findings in relevant primary human cells are still required to foster clinical translation of genetic engineering.

We have shown that both AAV and LV transduction activate DDR in human HSPC [23] (Fig. 2). This activation is mediated by p53 and occurs in absence of an integration-dependent DSB. The strength of this response correlates with the amount of vector-derived DNA that reaches the nucleus of the transduce cell [23]. Our results point towards a dose-dependent mechanism sensing the presence of extra-chromosomal DNA within the nucleus that readily translates into broader DDR in human HSPC. The peak of p53 activation usually occurs 48 h after transduction and tends to normalize 5 days post transduction. This signalling is mediated by the apical ATM kinase and seems very specific to human HSPC [23]. Indeed, murine Lin^-^ HSPC did not show signs of DDR upon LV or γRV transduction but activated a robust type I IFN response instead. It is possible that the same sensors activate distinct pathways in mouse and human cells. Importantly, vector-mediated activation of DDR lead to a mild but significant increase in apoptosis and decreased cell proliferation of human HSPC in culture [23]. These in vitro functional consequences translate in vivo in a lower engraftment of transduced HSPC, in particular during the early phases of haematopoietic reconstitution. In agreement with our observations, p53-dependent DDR was confirmed in the context of gene editing in human HSPC [79] and other stem cell types [80]. Although DSB-inducing gene editing components such as ZFN or CRISPR triggered DDR in HSPC, in particular when using low-specificity reagents, most of the p53 response could be ascribed to the presence of AAV in this context [79]. The functional consequences observed in this study were similar to those observed upon LV and AAV transduction alone, including cell cycle delay and a twofold lower engraftment of edited cells in vivo. Nevertheless, the single contributions of vector signalling and DSB induction by the editing machinery to the in vivo phenotype remain to be investigated in this setting. Of note, Cromer et al. reported low impact of AAV6 transduction per se on the transcriptome of human CD34^+^ cells, with electroporation of Cas9 mRNA and sgRNA being major drivers of antiviral and low DDR responses [81]. Differences between this study and ours could reside in different technological platforms for transcriptome analysis, with microarray techniques being less sensitive respect to bulk RNAseq, or to different time points analysed.

While gene editing mediated induction of DDR seems a logical consequence of the DSB generated by the site-specific nucleases within the genome, activation of such pathways in absence of physical breaks to the genomic DNA specifically in human HSPC was less expected. The yet to be identified factors involved in this DSB-independent recognition of exogenous DNA may converge with known DSB sensors or represent a novel link between nucleic acid sensing and DDR in HSPC. Of note, both LV and AAV can evade canonical antiviral innate immune sensing in HSPC [23]. In this regard, HIV has evolved several mechanisms of immune evasion that include exploiting host factors stabilizing the viral capsid prior to nuclear import to hide its genetic material from the many cytosolic sensors [51], and taking advantage of host endonucleases to clear excess RT products [82]. Similar mechanisms may exist for AAV that uncoats directly in the nucleus [83] (Fig. 2). It will also be of interest to evaluate whether some of the viral proteins capable of antagonizing innate and DDR sensors could be harnessed to render HSPC gene engineering more stealth.

## Restriction factors

**Fig. 3:**
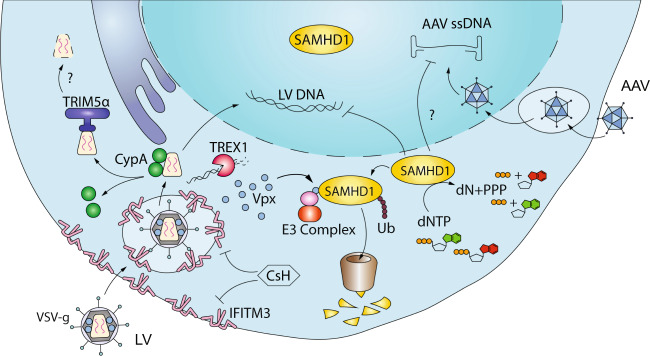
LV and AAV restriction factors. VSV-g pseudotyped LV suffer from IFITM3-mediated restriction that can be overcome by CsH during transduction. AAV are non-enveloped vectors insensitive to IFITM3 despite their endocytic entry route. After nuclear entry, LV exploit several host factors such as CypA and TREX1 to evade innate immune radars and capsid degradation by TRIM5α or TRIMCyp. During reverse transcription and dsDNA synthesis, both LV and AAV require dNTP. Thus SAMHD1, a master dNTP regulator, could restrict both vectors. The lentiviral accessory protein Vpx can efficiently target SAMHD1 for proteasomal degradation, relieving the block in DNA synthesis.

Because LV rely on the same cellular machinery as HIV-1 to reach the nuclear compartment of target cells and integrate within the host genome, they likely remain vulnerable to the host antiviral responses targeting the early steps of the viral life cycle in this context [84]. This may be of particular relevance in HSPC as they harbour high intrinsic expression of antiviral ISG [85], which may potentially contribute to their resistance to viral vector-based gene therapy approaches. Moreover, pseudotyped envelop glycoproteins and absence of all the accessory and regulatory viral proteins (Vpr/Vpx, Vpu, Vif, Nef, Tat, and Rev) may impact which host factor the vector will encounter and what means it will have to evade them. As restriction factors inhibiting infectious HIV-1 have been extensively reviewed elsewhere [86], we will discuss here those that have been reported to inhibit LV and that might be relevant also for AAV and genome editing, with a particular focus on HSPC (Fig. 3).

Interferon-induced-transmembrane protein 3 (IFITM3) inhibits a wide range of viruses including Ebola, Dengue, SARS, HIV, Influenza A, West Nile, and VSV [87–90]. The precise mechanism of action of IFITM3 remains elusive, but it depends on its subcellular localization and the viral envelope [91]. Recent evidence suggests that IFITM3 inhibits infection by altering the lipid composition and thus the physical properties of the cell membranes [92]. We have recently reported that IFITM3 blocks LV transduction in human and murine HSPC [93] (Fig. 3). The restriction imposed by IFITM3 occurs at the level of VSV-g-mediated entry, as MLV-derived amphotropic envelope glycoprotein or cytoplasmic tail mutants of the baboon envelope glycoprotein (BaEV-TR) pseudotyped LV remain resistant to IFITM3-mediated restriction. In agreement with our observation, I﻿FITM proteins can limit LV gene transfer to airway epithelia [94]. Interestingly, pseudotyping LV with the BaEV-TR has been suggested to significantly improve transduction efficiencies in human CD34^+^ cells compared to VSV-G pseudotyped vectors, including in unstimulated HSPC [95]. In the light of our findings, this advantage is likely explained, at least in part, by their capacity to bypass IFITM3 restriction. Since AAV does not possess an envelope, it remains insensitive to IFITM3 restriction [93], potentially contributing to its higher efficiency in HSPC gene editing as compared to LV-based donor DNA delivery strategies [3].

SAMHD1 is a well-known restriction factor for HIV in myeloid and resting T cells [96, 97]. Moreover, it has been shown to inhibit viral infection and LV transduction in human and murine myeloid cells [98, 99]. SAMHD1 acts as a tetramer to regulate the amount of dNTP in a cell by constantly degrading dNTP to dN [100] (Fig. 3). Consequently, in presence of SAMHD1, the limited dNTP pool impairs reverse transcription and the overall efficiency of transduction or infection is low. Importantly, since the restriction is based on the control of dNTP concentration, most of the SAMHD1 restriction is confined to non-proliferating cells, such as macrophages and dendritic cells. In agreement, the relevance of SAMHD1-mediated restriction in HPSC is minimal when these cells are stimulated with growth-promoting cytokines to allow cell survival and proliferation [101]. However, it is possible that SAMHD1 plays some role in restricting transduction of quiescent, unstimulated HSPC. Restriction factors such as IFITM3 that act prior to RT may also potentially mask some of its effects on LV transduction in HSPC. Of note, since also AAV require dNTP for dsDNA synthesis [102] the role of SAMHD1 restriction may be more pleiotropic than what previously thought (Fig. 3).

TRIM5α is a prototypic restriction factor that targets the incoming lentiviral capsids through ubiquitination and possibly, proteasomal degradation [103, 104]. This leads to a block in reverse transcription and a process that dismantles the virus. Although human TRIM5α has little or no antiviral activity against HIV-1 in human cells [103], recent evidence suggests that human TRIM5α can inhibit HIV if the protective interaction with the host factor CypA is disrupted [105] (Fig. 3). These effects may be cell-type specific, as we did not observed particular impairments in the transduction efficiencies of LV harbouring a capsid mutant no longer interacting with CypA in HSPC [52]. Commonly used γRV are usually derived from NB-tropic Moloney mouse leukaemia virus (Mo-MLV) and thus remain largely insensitive to human TRIM5α mediated restriction [106, 107]. On the other hand, TRIM5α (TRIM-Cyp) expressed in Old World and in some New World monkeys efficiently restricts HIV-1 but not SIV infection [108]. These species-specific aspects of cell intrinsic immunity become particularly important when choosing non-human primate models for preclinical gene therapy studies.

Less is known for the restriction factors that specifically target AAV life cycle as the wild-type virus is not pathogenic to humans and studies on AAV–host interactions have been limited. However, it has been shown that proteasome inhibitors can increase AAV2 transduction human cell lines (Hela, HepG2) [109]. In addition, AAV2 and AAV5 capsids are a substrate for ubiquitin conjugation, potentially tagging them for proteasome-dependent degradation [110, 111]. Since various restriction factors act by targeting for degradation portions of incoming viruses, it is tempting to speculate that a similar mechanism may counteract and potentially restrict AAV in HSPC. Interestingly, proteasome inhibitors enhance LV transduction in HSPC [112], raising the possibility that some yet to be identified antiviral factors can limit transduction across vector types.

## How to overcome cell intrinsic hurdles to gene engineering in HSPC

A better understanding of the relationship between commonly used viral vectors and their target cells can guide the development of optimized gene therapy strategies. In particular, restriction factors and nucleic acid sensors described above can be counteracted pharmacologically or by modifying viral vectors to accommodate antagonizing accessory proteins to increase efficiency and safety of several gene therapy applications (Figs. 1–3).

AAV transduction can be increased in vivo by transiently inhibiting TLR9 through an antagonizing ODN that could potentially be integrated within the AAV genome to prevent innate immune signalling [28] (Fig. 1). In the context of integrating vectors and ex vivo gene manipulation, a number of transduction enhancers have been developed and compounds such as 16,16-dimethyl prostaglandin E_2_ (PGE_2_) and LentiBOOST™ are already included in clinical trials [113]. For most of these enhancers, the molecular mechanisms leading to their biological effects on transduction efficiency are still unclear and may represent a potential concern in terms of long-term safety. PGE_2_ has been shown to relieve an early, still unidentified, transduction block and increase gene transfer efficiency roughly by twofold [11, 114]. However, reports of loss of primitive HSPC due to PGE_2_ exposure are raising concerns regarding the long-term benefits in patients [115] and underscore the importance of better characterizing the enhancer mechanisms and potential side effects these compounds may have on HSPC biology. Transduction enhancers such as poloxamers [116] or the polycationic protamine sulfate [117] act by favouring vector-cell contact, potentially through fluidifcation of the vector and cellular lipid membranes [116, 118]. The combination of LentiBOOST™ with protamine sulfate has been reported to yield sixfold higher LV transduction efficiency with minimal impact on HSPC biology in vitro [116]. Whether such effects will persist in the long-term repopulating HSPC in vivo remains to be determined, as significant drops in vector marking have been observed between the in vitro drug product and long-term repopulating cells in patients in most of the gene therapy trials conducted thus far, including those in which transduction enhancers have been employed [5, 6, 60, 113, 119, 120].

We have recently uncovered a way to overcome IFITM3-mediated restriction in HSPC by adding Cyclosporine H (CsH) during the ex vivo transduction phase [93] (Fig. 3). CsH transiently downregulates IFITM3 in HSPC thus leading to an increase in LV entry efficiency. CsH yields a 5–10-fold increase in transduction efficiency in long-term repopulating HSPC in vivo, rendering it the most potent enhancer of HSPC gene transfer described thus far [11, 93]. Importantly, CsH does not impact viability nor engraftment capacity of human HSPC, conversely to other enhancers such as Rapamycin (Rapa) or Cyclosporine A (CsA) that have shown some degree of toxicity, in particular in the clinically relevant mPB-derived HSPC [121]. Indeed, cell-source dependent effects of enhancers should be carefully evaluated as significant differences may exist with cord-blood and BM-derived HSPC being usually less sensitive as compared to mPB-derived CD34^+^ cells [121]. Of note, CsH significantly enhances also non-integrating LV-based gene editing as it increases the donor DNA template availability in HPSC [121]. This is of particular interest in settings in which larger donor DNA cassettes that AAV is unable to accommodate are required.

Interestingly, also Rapa has recently been suggested to increase transduction through transient depletion of IFITM3 from human HSPC [122]. However, the fold enhancement conferred by Rapa remains inferior to CsH and their combination is additive [93], suggesting distinct mechanisms of actions for the two compounds. Since AAV is not sensitive to IFITM3 restriction, CsH does not enhance AAV transduction efficiency in HSPC [93]. Recently, cyclic resveratrol trimer caraphenol A has been reported to alter the amount of IFITM2 and IFITM3 on late endosome and thus increase transduction efficiency in HSPC [123]. How these compounds compare or add to CsH in the context of clinically relevant HSPC sources and vectors remains to be investigated. IFITM3 restriction could in principle be overcome also by IFITM3-resistant envelopes such as Baev-TR [93]. However, the overall poor vector titre usually characterizing non-VSV-g pseudotyped LV render them difficult to translate as the overall gene marking will unlikely reach levels sufficient for clinical benefit. Combinations of different enhancers usually yield further benefit in terms of gene marking levels. Careful evaluation of toxicity and long-term efficacy becomes crucial in this context as multiple, often poorly characterized pathways are simultaneously affected.

The natural capacity of viral accessory proteins to counteract restriction factors can be harnessed to overcome host restriction in gene therapy. For example, the HIV-2 and SIV accessory protein Vpx targets SAMHD1 for proteasomal degradation [97–99]. When Vpx is present in the viral particle SAMHD1 is readily degraded and the dNTP in the cell rise to levels that increase efficiency of reverse transcription and transduction [97–99]. HIV-1 does not normally encode nor package Vpx into virions but the p6 portion of the Gag polyprotein in the LV packaging construct can be substituted with the one derived from SIV to allow incoportation of Vpx [99] (Fig. 3). Vpx incorporation does not affect vector titre but renders LV highly efficient in transducing primary human myeloid cells [99]. This strategy has already been employed to increase the transduction of MDM and DC in relevant preclinical settings [124, 125] but only limited benefit has been reported in HSPC thus far [101]. Coupling Vpx incorporation with early acting transduction enhancers could potentially be beneficial.

Donor variability remains a significant issue for the field as underscored also by the high variability in gene marking recently observed for β-Thalassemia gene therapy patients [5, 6]. Individual differences in innate immune factors governing permissiveness of HSPC to gene manipulation could contribute in this context. Interestingly, we have observed that variable IFITM3 protein levels negatively correlate with HSPC permissivity to transduction across donors [93]. In our study, the least permissive donors expressed the highest IFITM3 levels and benefitted most from CsH. These data suggest that CsH, besides enhancing transduction, may have the unique potential to mitigate donor variability during ex vivo gene therapy, a major benefit for the design of better-controlled clinical trials and, eventually, standardized medicinal products. Variations in expression levels of the antiretroviral host factor TRIM5α have also been suggested to correlate with HSPC permissiveness to lentiviral transduction [126] although human TRIM5α inhibition of HIV-1 is demonstrably weak [127]. It is possible that, differences in TRIM5α levels, and other ISGs, reflect variable IFITM3 levels rather than direct TRIM5α effects on transduction. Alternatively, expression of co-factors protecting LV from TRIM5α in human cells may also vary among individuals.

As our tools to enhance transduction become increasingly efficient, aspects related to dose-dependent vector signalling will need to be taken into consideration in designing optimized gene engineering protocols. Indeed, loss of gene marking has been observed with transduction enhancers at high vector doses [128], likely due to increased vector-mediated activation of DDR [23]. Among the drugs available to inhibit p53 activation upon LV and AAV transduction, the ATM inhibitor (ATMi) KU55933 lead to significant decrease in vector signalling, ex vivo apoptosis and rescued delayed HSPC engraftment kinetics in vivo, both in the context of gene addition and editing [23, 79] (Fig. 2). Approaches directly targeting p53 such as transient overexpression of GSE56, a dominant negative form of p53 [129], also prevent vector signalling and have been integrated in gene editing protocols to enable efficient engraftment of edited HSPC with no detectable effect on genotoxicity [23, 79] (Fig. 2). Transient inhibition of p53 in the bulk population of cells that undergo gene therapy could also decrease the probability of selecting pre-existing p53 mutant clones that are less sensitive to vector signalling during transduction or gene editing.

## Conclusions and perspectives

Taken together, we have summarized here some of the most significant examples of how dissecting the crosstalk occurring between HSPC and different gene manipulation platforms can inform the development of more stealth and efficient gene therapy approaches in the future. Although our review has focused mostly on nucleic sensing mechanisms in human HSPC, these principles likely apply with some differences also to other primary cells, such as T cells and monocytes, in the context of ex vivo manipulation as well as to some extent to in vivo genetic engineering approaches. There is still significant room for improvement as the gene manipulation toolbox and patient cohorts expand. In particular, evaluation of combinatorial approaches involving strategies that increase gene transfer or editing efficiency while at the same time dampening vector or nucleic acid-mediated signaling in HSPC will allow maximizing the potential of gene engineering. In addition, understanding vector and enhancer signalling in the context of patient cells will be critical. Specific gene defects or altered BM microenvironements can potentially affect HSPC responses to components of the gene engineering machinery, thus potentially requiring tailored protocols for specific disease settings. Studies on larger patient cohorts aimed at dissecting determinants of individual variability in gene therapy efficacy would help uniform and guarantee clinical benefits for all patients. Finally, very little is currently known about how novel delivery methods involving ssODNs, dsDNA, mRNA and modified mRNAs engage with host innate sensors and potentially affect cell fitness, warranting extensive investigation in the future. Overall, better understanding of the basic mechanisms of vector–host interactions in HSPC and other primary targets of gene therapy will help shed light on the molecular mechanisms of innate immunity, uncover novel immune barriers potentially hampering gene transfer and correction and deliver new knowledge critical for the development of improved vectors and gene engineering strategies in clinically relevant targets.
